# Fenretinide-dependent upregulation of death receptors through ASK1 and p38*α* enhances death receptor ligand-induced cell death in Ewing's sarcoma family of tumours

**DOI:** 10.1038/sj.bjc.6605896

**Published:** 2010-09-28

**Authors:** D E White, S A Burchill

**Affiliations:** 1 Candlelighter's Children's Cancer Research Group, Section of Experimental Oncology, Leeds Institute of Molecular Medicine, St. James's University Hospital, Beckett Street, Leeds, UK

**Keywords:** ASK1, death receptors, Ewing's sarcoma family of tumours, fenretinide, p38^MAPK^, TRAIL

## Abstract

**Background:**

Sustained p38^MAPK^ phosphorylation upregulates p75 neurotrophin (p75^NTR^) and induces apoptosis in Ewing's sarcoma family of tumours (ESFT). As fenretinide induces ESFT death through sustained p38^MAPK^ phosphorylation, we hypothesised that this may be effected through upregulation of death receptors (DRs) and that treatment of fenretinide plus DR ligands may enhance apoptosis.

**Methods:**

DR expression was determined by flow cytometry. Trypan blue exclusion assays, caspase-8 flow cytometry and immunoblotting for Bid were used to measure cell death.

**Results:**

Fenretinide upregulated cell surface expression of tumour necrosis factor-related apoptosis-inducing ligand (TRAIL) receptors, FAS and p75^NTR^, in an ASK1- and p38*α*-dependent manner. Cotreatment with fenretinide and DR ligands resulted in synergistic death compared with either agent alone; caspase-8 and Bid were cleaved in a time-dependent manner. Fenretinide did not increase DR expression in non-malignant cells. Furthermore, fenretinide, TRAIL or a combination of both agents was non-cytotoxic to non-malignant cells. Etoposide and actinomycin D increased expression of all DRs examined, whereas vincristine increased FAS alone. Only actinomycin D and TRAIL, and etoposide with TRAIL or FasL, enhanced death compared with either agent alone.

**Conclusion:**

The synergistic death observed with fenretinide and DR ligands suggests that this combination may be an attractive strategy for the treatment of ESFT.

Ewing's sarcoma family of tumours (ESFTs) arise in bone and soft tissue sites. ESFT occurs at all ages, although there is a peak incidence at 10–25 years of age. Recurrence and metastasis pose the greatest challenge for successful treatment of ESFT; patients in this cohort have a 20% or less 5-year disease-free survival rate (Proctor *et al*, 2009). There is therefore an urgent need for effective treatment strategies. Fenretinide, a synthetic retinamide with promising chemopreventive and chemotherapeutic properties, is well tolerated in both adult (Hail *et al*, 2006) and paediatric (Garaventa *et al*, 2003; Villablanca *et al*, 2006; Formelli *et al*, 2008) phase I clinical trials. We have previously demonstrated that fenretinide induces apoptosis through generation of reactive oxygen species (ROS) and phosphorylation of p38 mitogen-activated protein kinase (p38^MAPK^) in ESFT. Delayed growth of subcutaneous ESFT in nude mice was also observed following fenretinide treatment (Myatt *et al*, 2005).

The tumour necrosis factor (TNF) receptor (TNFR) superfamily includes a subclass of receptors known as death receptors (DRs), so called as they contain a cytoplasmic region called the death domain (DD) that is important for the induction of DR-mediated apoptosis. Members of the DR family include TNF-R1, p75 neurotrophin (p75^NTR^), FAS, DR4 and DR5 (Debatin and Krammer, 2004; Ashkenazi, 2008). Binding of TNF superfamily ligands to their cognate receptors induces receptor trimerisation and recruitment of Fas-associated death domain (FADD), which subsequently recruits procaspase-8 to form the death-inducing signalling complex (DISC). This association of proteins induces caspase-8 cleavage, which subsequently cleaves caspase-3 to induce a non-mitochondrial apoptotic pathway (Debatin and Krammer, 2004; Ashkenazi, 2008). Bid is a BH3-only member of the Bcl-2 family that is cleaved by caspase-8 in response to certain stimuli, whereby it translocates to the mitochondria to induce the oligomerisation of BAX or BAK and release of cytochrome C. In some cell types, sufficient quantities of caspase-8 are present to induce apoptosis independently of the mitochondria, whereas in cells with low levels of caspase-8, Bid cleavage is required to amplify death through mitochondria (Debatin and Krammer, 2004; Ashkenazi, 2008).

Tumour necrosis factor-related apoptosis-inducing ligand (TRAIL) binds to DR4 and DR5, and to three decoy receptors (DcRs) that either lack an intracellular DD in the case of DcR1 or contain a truncated DD in the case of DcR2. The third DcR, osteoprotegrin is a soluble receptor. These DcRs do not induce apoptosis because of the absence of a functional DD (Ashkenazi, 2008). As TRAIL is reported to be selective in inducing apoptosis in malignant cells, although sparing healthy cells (Ashkenazi, 2008), numerous proapoptotic receptor agonists have been developed that include recombinant TRAIL and monoclonal agonistic antibodies against DR4 and DR5. Many show promise in clinical trials, both as monotherapies and in combination with biological agents, including histone deacetylase inhibitors (HDACi), bortezomib and chemotherapeutics (Ashkenazi, 2008).

Ewing's sarcoma family of tumour cells and tumour samples express both TRAIL DR and FAS (Kontny, 2006), although p75^NTR^ has only been examined in cell lines (Westwood *et al*, 2002; Williamson *et al*, 2004). Approximately 80% of ESFT cell lines examined are reported to be sensitive to TRAIL-induced apoptosis (Kontny, 2006), and those that are resistant can be sensitised by pretreatment with interferon gamma (IFN*γ*; Kontny, 2006; Lissat *et al*, 2007). Enhanced death is also reported when ESFT cells are treated with TRAIL and bortezomib (Lu *et al*, 2008) or HDACi (Sonnemann *et al*, 2007). Recent analysis of the agonistic DR4 monoclonal antibody Mapatumumab in a paediatric preclinical testing program failed to demonstrate preclinical efficacy in subcutaneous ESFT models (Smith *et al*, 2009). However, the agonistic DR5 monoclonal antibody (M413; Amgen, Cambridge, UK) is reported to decrease ESFT growth following intramuscular injection (Merchant *et al*, 2004). Furthermore, delivery of TRAIL through non-viral gene therapy diminished tumour growth and increased animal survival in ESFT (A673) mouse xenografts (Picarda *et al*, 2010). A phase I trial is currently recruiting young patients with solid tumours or lymphomas refractory to conventional chemotherapeutics to assess the effects of the DR5 agonistic antibody Lexatumumab either alone or in combination with IFN*γ* (ClinicalTrials.gov Identifier: NCT00428272).

We previously demonstrated that sustained p38^MAPK^ phosphorylation is required for the upregulation of p75^NTR^ protein expression and induction of apoptosis by basic fibroblast growth factor (bFGF; Williamson *et al*, 2004). As fenretinide also induces ESFT apoptosis through sustained p38^MAPK^ phosphorylation (Myatt *et al*, 2005), we hypothesised that fenretinide-induced death may be effected in part through the induction of DR. If correct, we hypothesised that treatment with DR ligands plus fenretinide should amplify the initial apoptosis induced by fenretinide. Results indicate that fenretinide induced upregulation of DR at the cell surface in an apoptosis signal-regulating kinase (ASK) 1- and p38*α*-dependent manner. Furthermore, cotreatment with DR ligands and fenretinide resulted in synergistic death compared with either agent alone. However, only certain chemotherapeutic drugs (etoposide and actinomycin D) upregulated DR and this did not always occur in a p38^MAPK^-dependent manner or correspond with enhanced cell death when cotreated with drug and DR ligands.

## Materials and methods

### Cells and reagents

The cell lines used are described in Supplementary Table 1. All reagents were prepared and stored according to the manufacturer's instructions and obtained from the following suppliers: recombinant TRAIL and Fas ligand (FasL) from R&D systems (Abingdon, UK), *γ*-^32^P-ATP from GE Healthcare Life Sciences (Little Chalfont, UK). All other chemicals were obtained from Sigma (Poole, UK) unless stated otherwise. Fenretinide (gift from the National Cancer Institute), BAY 11-70892 (Calbiochem, Nottingham, UK) and SB202190 (Calbiochem) were prepared as previously described (Myatt *et al*, 2005; White and Burchill, 2008). BIRB0796 (10 mM stock in DMSO, stored at −20 °C) was purchased from Dr Hilary McLaughlan, University of Dundee. All antibody dilutions were determined empirically. Total and phosphorylation-specific p38MAPK antibodies, used at a dilution of 1 : 5000 and 1 : 10000, respectively, anti-MKK3 and anti-phospho-MKK3/6 (this antibody recognises a phosphorylated activation loop conserved between MKK3 and MKK6) antibodies, used at 1 : 500, and Bid antibody (1 : 500) were all purchased from Cell Signaling Technology (Danvers, MA, USA). Protein A/G PLUS-Agarose, antitubulin (1 : 5000) and anti-ASK 1 (H-300, 1 : 100) antibodies were purchased from Santa Cruz Biotechnology (Santa Cruz, CA, USA).

Antibodies for flow cytometry were diluted in FACS buffer (PBS, 1% fetal calf serum (SeraLab, Haywards Heath, UK), 0.1% sodium azide). FAS-FITC (Beckman Coulter, High Wycombe, UK) and IgG1-FITC (Dako Cytomation, Ely, UK) antibodies were used at 1 : 10 dilution. p75^NTR^ antibody (Promega, Southampton, UK) was used at 1 : 500. All TRAIL-R antibodies (Alexis Biochemicals, Exeter, UK) were used at 1 : 100 dilutions, except for DR4 (1 : 50). Secondary antibodies goat F(ab2) antirabbit IgG-PE (Caltag Laboratories, Paisley, UK) and goat F(ab)2 antimouse IgG-PE (Southern Biotechnology Associates from Cambridge Biosciences, Cambridge, UK) were used at 1 : 200.

### Cell surface expression of receptors

Cells were harvested, washed in FACS buffer and incubated with antibodies (4 °C, 30 min for both primary and secondary antibodies). Controls included secondary antibody only (TRAIL receptors and p75^NTR^) or IgG1-FITC antibody (FAS). Cells were washed, resuspended in 300 *μ*l FACS buffer and cell surface expression was determined by flow cytometry (FACSCalibur, BD, Oxford, UK). Data were analysed using CellQuest software (BD Biosciences, Oxford, UK). Results are presented as (mean of the median fluorescence intensity for each DR minus median fluorescence with secondary (TRAIL and p75^NTR^) or IgG1-FITC (FAS) antibody)±s.e.m. (*n*=9). The following cell lines and treatments were used as positive controls to optimise antibodies (data not shown): jurkat cells (DR5), A375 cells (DR4), MCF-7 cells (DcR2), doxorubicin (500 ngml^−1^, 15 h)-treated MCF-7 cells (DcR1 and FAS) and bFGF (20 ngml^−1^, 16 h)-treated TC-32 cells (p75^NTR^).

### Fenretinide array

RNA was extracted from both untreated and fenretinide (3 *μ*M, 8 h)-treated TC-32 cells using Ultraspec (Biotecx, Houston, TX, USA), as previously described (Brownhill *et al*, 2007). Total RNA (2 *μ*g) was used to generate a biotin-labelled apoptosis-specific cDNA library, which was hybridised with a GEArray Q-series human apoptosis array (SA Biosciences, Frederick, MD, USA), according to the manufacturer's instructions. Arrays were processed for chemiluminescence, data were extracted with ScanAlyze software (Eisen Lab, Berkeley, CA, USA) and analysed using the GEArray analyser software (SA Biosciences).

### Electroporation of TC-32 cells with small interfering RNA (siRNA)

Cells were electroporated as described previously (Myatt *et al*, 2005; White and Burchill, 2008) with 500 nM siRNA directed against ASK1 (Santa Cruz Biotechnology; a pool of three individual ASK1 siRNAs) or scrambled siRNA (Silencer Negative Control #1; Ambion, Huntington, UK). Sequences of all siRNAs are company propriety. ASK1 knockdown was confirmed by quantitative real-time RT–PCR using ASK1 Taqman Gene Expression Assay ID: Hs00178726 (Applied Biosystems, Warrington, UK) and *β*2 M probes and primers, as described previously (Brownhill *et al*, 2007). Electroporated cells were analysed after 48 h for receptor expression by flow cytometry or viable cell number using the Trypan blue exclusion assay.

### ASK 1 immunecomplex kinase assay

Fenretinide (3 *μ*M, 0–60 min)-treated cells were lysed in buffer (20 mM Tris-HCl (pH 7.5), 150 mM NaCl, 1 mM EDTA, 1 mM EGTA, 1% Triton X-100, 2.5 mM sodium pyrophosphate, 1 mM *β*-glycerol phosphate, 1 mM Na_3_VO_4_, 1 *μ*g/ml leupeptin and 1 mM phenylmethylsulphonyl fluoride) on ice for 5 min. Lysates were clarified by centrifugation (10 000 **g**, 15 min, 4 °C) and protein concentration was determined using the Bio-Rad DC protein assay (Bio-Rad Laboratories, Hemel Hempstead, UK). Total protein lysate (200 *μ*g) was incubated with anti-ASK1 antibody (2 *μ*g, 1 h), and then incubated with 20 *μ*l protein A/G-sepharose overnight at 4 °C with rotation. Beads were collected by centrifugation (4000 **g**, 3 min, 4 °C) and washed twice with 500 *μ*l lysis buffer and kinase buffer (25 mM Tris (pH 7.5), 5 mM *β*-glycerolphosphate, 2 mM DTT, 0.1 mM Na_3_VO_4_ and 10 mM MgCl_2_). Pellets were resuspended in 50 *μ*l kinase buffer containing 200 *μ*M ATP, 2 *μ*Ci *γ*-^32^P-ATP and 4 *μ*g myelin basic protein (MBP), and incubated at 30 °C for 30 min. Reactions were terminated by adding 25 *μ*l 2 × SDS loading buffer. Samples were heated at 95 °C for 5 min and separated by SDS–PAGE. The gel was rinsed in water, stained in Bio-safe Commassie (Bio-Rad Laboratories) for 1 h and dried under vacuum. ASK1 activity was assessed by incorporated ^32^P in MBP, as determined by autoradiography, and quantified using phosphorimager (Quantity One software, Bio-Rad Laboratories). SH-SY5Y cells treated with or without 6-hydroxydopamine (6-OHDA; 100 *μ*M, 1 h) served as positive and negative controls, respectively.

### Cell viability

Viable cell number was determined using the Vi-cell Trypan blue exclusion assay (Beckman Coulter) as described previously (White and Burchill, 2008).

### Caspase-8 activity

Caspase-8 activation was determined using the fluorochrome inhibitor of caspases (FLICA) FAM-LETD-FMK apoptosis detection kit (Immunochemistry Technologies, Bloomington, MN, USA) according to the manufacturer's instructions. The FLICA inhibitor covalently binds to a reactive cysteine residue on the large subunit of active caspase-8 heterodimer. This inhibits any further caspase activity and retains FLICA intracellularly so that active caspase-8 molecules can be quantified by flow cytometry. Jurkat cells treated with or without etoposide (30 *μ*M, 16 h) served as positive and negative controls, respectively.

### Immunoblotting

Lysates were extracted and proteins detected by immunoblotting and visualised using the Odyssey infrared imaging system (Li-cor, Lincoln, NE, USA) as described previously (Myatt *et al*, 2005).

### Statistical analyses

Statistical analyses were undertaken using GraphPad Prism 5 or SAS 9. Data were analysed by analysis of variance and a Bonferroni *post hoc* test when comparing three or more conditions. An interaction effect between drug and DR ligand was assessed to determine whether enhanced death had occurred. An unpaired two-tailed *t*-test was used to analyse differences in untreated versus fenretinide-treated non-malignant cells. Variations between means were considered significantly different at *P*⩽0.05.

## Results

### Fenretinide upregulates DR and DcR in ESFT cells

**Figure 1:**
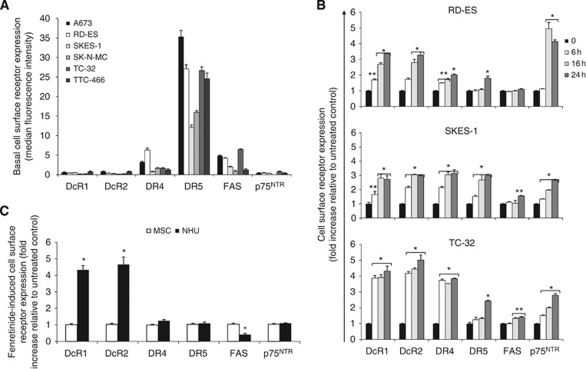
Fenretinide upregulates DR and DcR in ESFT cells. (**A**) Basal cell surface expression of DcR and DR was determined by antibody labelling cells and by analysing levels by flow cytometry. Results are presented as the mean of the median fluorescence intensity for each DR minus median fluorescence with secondary (TRAIL and p75^NTR^) or IgG1-FITC (FAS) antibody±s.e.m. (*n*=9). (**B**) ESFT cells were treated with fenretinide (3 *μ*M, 0–24 h) or vehicle control for 0, 6, 16 or 24 h and (**C**) NHUC and MSC were treated with fenretinide (3 *μ*M) for 24 h. Receptor cell surface expression was examined by antibody labelling and flow cytometry. Results are presented as the fold increase of the mean of the receptor median fluorescence intensity relative to untreated control samples±s.e.m. (*n*=6). ^*^P⩽0.001, ^**^P⩽0.05.

Basal cell surface expression of TRAIL receptors, FAS and p75^NTR^, was established in a panel of ESFT cell lines. DR5 was the most abundantly expressed receptor in all cell types, whereas DR4 was expressed at much lower levels (Figure 1A). IgG1-FITC antibody was moderately expressed in all cells, except in SK-N-MC and TTC-466, with the highest expression detected in TC-32 cells. DcR1, DcR2 and p75^NTR^ were marginally expressed in ESFT cells. RD-ES, SKES-1 and TC-32 were selected for further studies because of their heterogeneity of receptor expression and the genotypic differences described previously (Westwood *et al*, 2002; Brownhill *et al*, 2007). Neither DR nor DcR were expressed in the non-malignant primary normal human urothelial cells (NHUC) or mesenchymal stem cells (MSC, data not shown).

We previously demonstrated that bFGF-induced apoptosis is dependent on p38^MAPK^-mediated induction of p75^NTR^ in ESFT cells (Westwood *et al*, 2002; Williamson *et al*, 2004). As fenretinide-induced apoptosis also requires sustained p38^MAPK^ phosphorylation (Myatt *et al*, 2005), we hypothesised that DR upregulation may be involved in fenretinide-induced death. Fenretinide (3 *μ*M, 8 h) strongly upregulated DR5 cDNA and moderately increased DR4 and DcR2 cDNA in TC-32 cells (Supplementary Figure 1 and Supplementary Table 2). No changes in the expression of other DR pathway components (DcR1, FAS, caspase-8, caspase-10, TRAIL, FasL and FADD) were observed.

The effect of fenretinide on cell surface receptor expression was also examined. Fenretinide induced rapid upregulation of both DcRs in all ESFT cells (Figure 1B, four- to five-fold in TC-32 cells, two- to four-fold in RD-ES and SKES-1 cells, *P*⩽0.001). DR4 was upregulated in a time-dependent manner in RD-ES and SKES-1 cells, and was strongly upregulated in TC-32 cells at 6 h, with no further increase observed up to 24 h. DR5 was increased at 16 h in SKES-1 cells and at 24 h in RD-ES and TC-32 cells (*P*⩽0.01). Fenretinide increased FAS expression from 16 h in TC-32 cells (*P*⩽0.01) and at 24 h in SKES-1 cells (*P*⩽0.05) but had no effect on FAS expression in RD-ES cells. p75^NTR^ expression was maximally increased in RD-ES cells at 16 h (4.9-fold), whereas a time-dependent increase occurred in SKES-1 and TC-32 cells (2.7-fold and 2.8-fold, respectively, at 24 h, *P*⩽0.001). Fenretinide (3 *μ*M, 24 h) did not affect DR4, DR5 or p75^NTR^ expression in NHUC (Figure 1C). However, it did induce a 2.5-fold downregulation of FAS and upregulation of both DcR1 (4.4-fold) and DcR2 (4.7-fold, *P*⩽0.001). Fenretinide had no effect on receptor expression in MSC, suggesting that fenretinide-induced DR upregulation is selective to cancer cells.

### Fenretinide-induced upregulation of DR is dependent on phosphorylation of ASK1 and p38*α*

**Figure 2:**
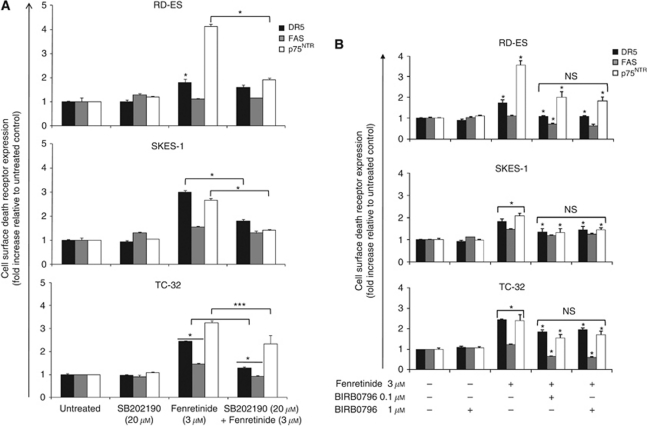

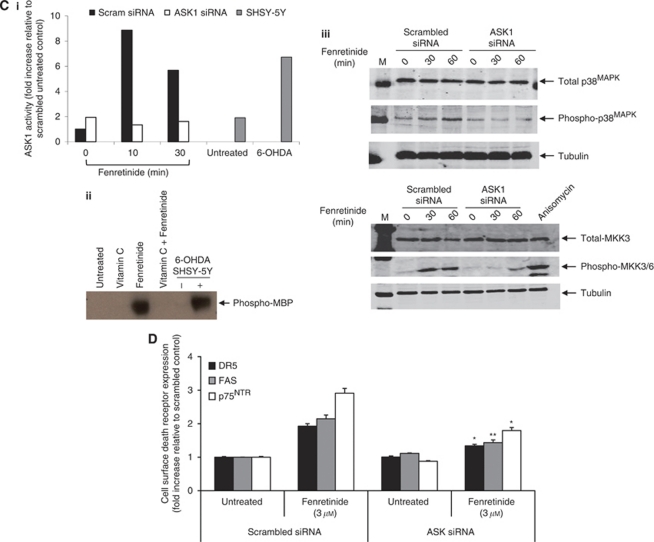
Fenretinide upregulation of DR is dependent on phosphorylation of ASK1, and p38*α*. Ewing's sarcoma family of tumour cells were pretreated with (**A**) SB202190 (20 *μ*M, 1 h) or (**B**) BIRB0796 (0.1 or 1 *μ*M, 24 h) before fenretinide treatment (3 *μ*M, 24 h) and DR expression was determined by antibody labelling and flow cytometry. Results are presented as the fold increase of the mean of the receptor median fluorescence intensity relative to untreated control samples±s.e.m. (*n*=6). (**Ci**) TC-32 cells were electroporated with scrambled or ASK1 siRNA (500 nM), and ASK1 kinase activity was assessed 48 h later by immunecomplex kinase assays using MBP as the substrate. Samples were subsequently resolved by SDS–PAGE and gels were analysed by quantitative autoradiography using a PhosphorImager system. Untreated and 6-OHDA (100 *μ*M, 1 h)-treated SH-SY5Y cells served as negative and positive controls, respectively, for ASK1 kinase activity. Graph shows ASK1 activity calculated as fold increase over untreated scrambled siRNA cells. (**Cii**) TC-32 cells were pretreated with vitamin C (100 *μ*M, 1 h) before fenretinide treatment (3 *μ*M, 10 min) and ASK1 immunecomplex kinase assays were performed. (**Ciii**) TC-32 cells were electroporated with scrambled or ASK1 siRNA (500 nM). Protein expression was determined 48 h later by immunoblot analysis for total p38^MAPK^ and phospho-p38^MAPK^ and total MKK3 and phospho-MKK3/6. Anisomycin (25 *μ*g/ml, 30 min)-treated TC-32 cells served as a positive control for MKK3/6 phosphorylation. Equal protein loading was confirmed by hybridisation to tubulin. M=molecular weight markers. Representative images from three independent experiments are shown. (**D**) TC-32 cells were electroporated with scrambled or ASK1 siRNA (500 nM) and were treated with fenretinide (3 *μ*M, 24 h) 24 h after electroporation. DR expression was determined by antibody labelling and flow cytometry, and results presented as the fold increase of the mean of the receptor median fluorescence intensity relative to untreated control samples±s.e.m. (*n*=6). ^*^P⩽0.001, ^**^P⩽0.01, ^***^P⩽0.05.

We examined the hypothesis that fenretinide-induced DR upregulation may be effected through p38^MAPK^ using the pharmacological inhibitor SB202190. SB202190 (20 *μ*M, 1 h) and fenretinide cotreatment (3 *μ*M, 24 h) decreased cell surface expression of DR5 in SKES-1 and TC-32 cells, of FAS in TC-32 cells and of p75^NTR^ in all cell types, compared with fenretinide-induced DR upregulation (Figure 2A, *P*⩽0.05: p75^NTR^ in TC-32 cells, *P*⩽0.001: other receptors and cell types). SB202190 alone had no effect on DR expression. These observations suggest that upregulation of some DRs by fenretinide may occur in a p38^MAPK^-dependent manner.

BIRB0796 was used to delineate which p38^MAPK^ isoform was required for fenretinide-induced DR upregulation; BIRB0796 inhibits p38*α* at 0.1 *μ*M, whereas 1 *μ*M inhibits all four isoforms (Kuma *et al*, 2005). BIRB0796 pretreatment (0.1 or 1 *μ*M, 24 h) before fenretinide treatment (3 *μ*M, 24 h) suppressed fenretinide-induced upregulation of DR5, p75^NTR^ and FAS in all cell types (Figure 2B, *P*⩽0.001). Although BIRB0796 treatment alone had no effect on DR expression, BIRB0796 and fenretinide cotreatment significantly decreased basal FAS expression in RD-ES cells. Furthermore, no significant difference was observed between the effects of 0.1 and 1 *μ*M of BIRB pretreatment, indicating that p38*α* is the isoform most likely to be required for fenretinide-induced DR upregulation.

Apoptosis signal-regulating kinase 1 is a redox-regulated MAPK kinase that regulates stress-activated MAPK in response to apoptotic stimuli (Coulthard *et al*, 2009). The effect of fenretinide on ASK1 kinase activity was determined by immunecomplex kinase assays using MBP as a substrate. Preliminary data indicated that in all ESFT cell lines examined (RD-ES, SKES-1 and TC-32), fenretinide (3 *μ*M) induced ASK1 kinase activity from 5 min, with maximium activation observed at 10 min. MBP phosphorylation decreased with time but remained above basal levels for up to 60 min (data not shown). To confirm whether ASK1 was an upstream regulator of p38^MAPK^-dependent fenretinide-induced DR upregulation, siRNA was used. ASK1 siRNA (500 nM, 48 h) reduced ASK1 mRNA levels by 79% in TC-32 cells compared with scrambled control cells (Supplementary Figure 2). Fenretinide (3 *μ*M) induced ASK1 kinase activity in scrambled siRNA-treated TC-32 cells and MBP phosphorylation was abolished in ASK1 siRNA-treated TC-32 cells (Figure 2Ci). Phosphorylation was comparable to that detected in the positive control SHSY-5Y neuroblastoma cells treated with 6-OHDA (100 *μ*M, 1 h). Furthermore, no ASK1 activity or p38^MAPK^ phosphorylation was detected in SHEP1 neuroblastoma cells (data not shown), a cell line that we have previously demonstrated to be resistant to fenretinide (Myatt *et al*, 2005; White and Burchill, 2008). Pretreatment of TC-32 cells with vitamin C (100 *μ*M, 1 h) before fenretinide treatment (3 *μ*M, 10 min) completely abolished ASK1 activity (Figure 2Cii; data not shown for SKES-1 and RD-ES cells), suggesting that fenretinide-induced ROS production is upstream of ASK1 activity in ESFT cells.

Immunoblot analysis identified fenretinide-induced p38^MAPK^ phosphorylation in scrambled siRNA-treated ESFT cells at 30 min. However, lower phosphorylation levels were observed in ASK1 siRNA-treated cells in response to fenretinide (Figure 2Ciii: representative image shown for TC-32 cells, data not shown for SKES-1 and RD-ES cells), thus confirming that p38^MAPK^ is downstream of ASK1 in the fenretinide-induced apoptotic pathway. A similar experimental design was used to identify the intermediate regulatory MAPK kinase (MKK3 or MKK6; Coulthard *et al*, 2009) between ASK1 and p38^MAPK^ in the fenretinide-induced apoptotic pathway. MKK3/6 was robustly phosphorylated 30–60 min after fenretinide treatment in scrambled siRNA-treated ESFT cells (Figure 2Ciii; representative image shown for TC-32 cells, data not shown for SKES-1 and RD-ES cells). This was a comparable level of phosphorylation to that detected in anisomycin-treated (25 *μ*g, 30 min) TC-32 cells used as a positive control. Lower levels of MKK3/6 phosphorylation were detected in ASK1-treated siRNA cells, suggesting that these MAPKK are downstream of ASK1 in the fenretinide-induced apoptotic pathway. No changes in total MKK3 or MKK6 expression were observed in either cell type.

Fenretinide induced DR upregulation in scrambled siRNA-treated TC-32 cells (Figure 2D), consistent with previous observations in unelectroporated cells (Figure 1B). However, reduced DR levels were observed in ASK1 siRNA-treated cells compared with scrambled siRNA cells, indicating that ASK1 is required for fenretinide-induced upregulation of DR (*P*⩽0.001: DR5 and p75^NTR^, *P*⩽0.01: FAS). Furthermore, ASK1 siRNA rescued fenretinide-induced death by 52% compared with scrambled siRNA cells (*P*⩽0.01, Supplementary Figure 3), demonstrating that fenretinide induces DR upregulation and cell death in an ASK1-p38^MAPK^-dependent manner in ESFT cells.

### Fenretinide sensitises ESFT cells to the DR apoptotic pathway to enhance cell death

**Figure 3:**
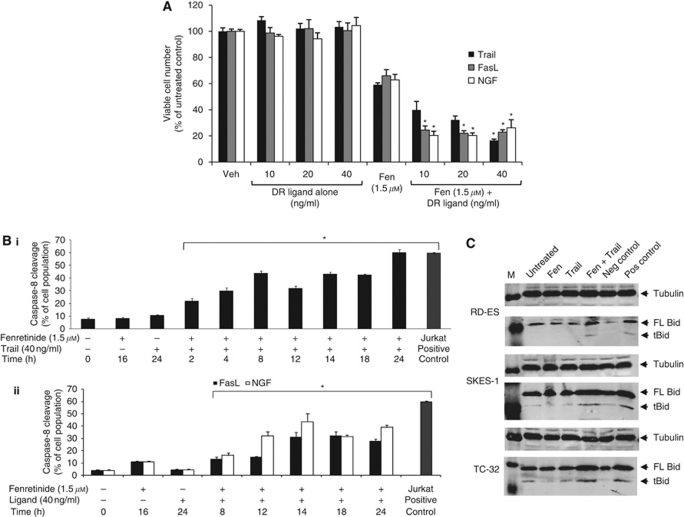
Fenretinide sensitises ESFT cells to the DR apoptotic pathway to enhance cell death. (**A**) TC-32 cells were treated with fenretinide (Fen; 1.5 *μ*M, 16 h) or vehicle control (Veh; ethanol treated cells), media were changed and cells were subsequently treated with the DR ligands TRAIL, FasL or NGF (0–40 ng/ml, 24 h). Viable cell number was determined by the Trypan blue exclusion assay. Results are presented as the mean of the viable cell number calculated as a percentage of untreated control cells±s.e.m. (*n*=9). Statistics indicate significant interactions between fenretinide and DR ligands, which is indicative of enhanced cell death; ^*^P⩽0.001. TC-32 cells were pretreated with fenretinide (1.5 *μ*M, 16 h) before DR ligands (40 ng/ml, 0-24 h) and caspase-8 activity was detected by flow cytometry. (**Bi**) Effect of fenretinide and (**Bi**) TRAIL, (**Bii**) FasL or NGF time course of caspase-8 cleavage in TC-32 cells. Results are presented as the mean of the percentage of cells with cleaved caspase-8, as detected within the M1 region±s.e.m. (*n*=9), ^*^P⩽0.001. Untreated and etoposide (30 *μ*M, 24 h)-treated Jurkat cells served as negative and positive control samples, respectively. (**C**) Total protein lysates from ESFT cells pretreated with fenretinide (Fen, 1.5 *μ*M, 16 h) before TRAIL treatment (40 ng/ml, 24 h) were examined for both full length (FL, 22 kDa) and truncated (cleaved) bid (tBid, 15 kDa) by immunoblotting. Equal protein loading was confirmed by hybridisation to tubulin. M=molecular weight markers. Negative (Neg) control=untreated Jurkat cells, positive (Pos) control=etoposide-treated (25 *μ*M, 16 h) Jurkat cells. Immunoblots are representative from three independent experiments.

As increased cell surface expression of DR would increase receptor availability to cognate ligands, we hypothesised that cotreatment of ESFT cells with fenretinide plus DR ligands (TRAIL for DR5, nerve growth factor (NGF) for p75^NTR^ and FasL for FAS) may enhance death. Treatment with DR ligands (0–40 ng ml^−1^, 24 h) alone did not decrease viable cell number (Figure 3A; we have performed viability assays with up to 400 ngml^−1^ TRAIL in six ESFT cell lines and have not observed cell death, unpublished observation). However, the combination of fenretinide and DR ligands resulted in synergistic cell death (reductions were 43% for TRAIL and NGF and 36% for FasL with 40 ng ml^−1^ DR ligand and 1.5*μ*M fenretinide) compared to fenretinide alone (Figure 3A, statistical interactions *P*⩽0.001) in TC-32 cells. Furthermore, neither DR ligands (40 ng ml^−1^, 24 h) nor fenretinide (3 *μ*M, 16 h) or a combination of both agents had any effect on viable cell number in non-malignant NHUC or MSC (data not shown; mean viable cell number calculated as percentage of untreated control ranged from 95±4% to 107±4%).

We next examined whether this enhanced death was effected through caspase-8 cleavage. No significant cleavage was observed following single treatments with fenretinide or DR ligands (Figure 3B and Supplementary Figure 4), supporting the hypothesis that fenretinide-induced apoptosis is effected through the mitochondrial death cascade (Myatt *et al*, 2005), and consistent with the observation that DR ligands do not reduce TC-32 viable cell number. However, combined treatment of fenretinide and TRAIL increased caspase-8 activity in a time-dependent manner, with 68% activity observed at 24 h (Figure 3Bi and Supplementary Figure 4, *P*⩽0.001). FasL and fenretinide induced 31% caspase-8 activation within 14 h exposure, which did not further increase up to 24 h, whereas NGF and fenretinide induced caspase-8 activity at 12 h (31%), with a further increase observed at 24 h (Figure 3Bii, *P*⩽0.001).

Immunoblot analysis demonstrated that fenretinide and TRAIL induced cleavage of Bid to generate a truncated form (tBid; Figure 3C) compared with untreated cells and cells treated with either agent alone. This was observed in all ESFT cell lines examined; cleavage was comparable to that in etoposide (25 *μ*M, 16 h)-treated Jurkat cells (positive control). These data indicate a putative role of truncated Bid in amplifying the DR pathway of apoptosis through the mitochondria in ESFT cells.

### Specific chemotherapeutics upregulate DR and enhance ESFT cell death

**Figure 4:**
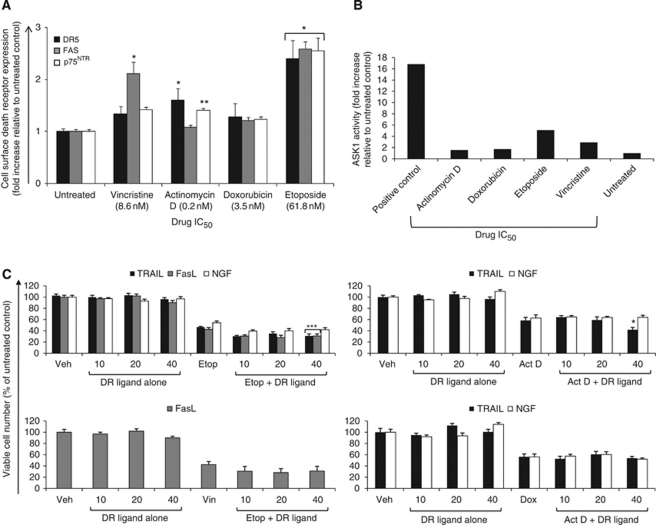
Specific chemotherapeutics upregulate DR and enhance ESFT cell death. (**A**) TC-32 cells were treated with the indicated drug IC_50_ or vehicle control for 48 h and cell surface DR expression was determined by antibody labelling and flow cytometry. Results are presented as the fold increase of the mean of the receptor median fluorescence intensity relative to untreated control samples±s.e.m. (*n*=6). ^*^P<0.001, ^**^P=0.03. (**B**) TC-32 cells were treated with chemotherapeutic agents (drug IC_50_, 48 h) or vehicle control, and ASK1 immunecomplex kinase assays were performed as described in figure legend **2C**. Positive control=6-OHDA (100 *μ*M, 1 h)-treated SH-SY5Y cells. Graph depicts ASK1 kinase activity presented as fold increase of drug-treated samples over untreated control, as quantified by phosphorimager analysis. (**C**) TC-32 cells were treated with chemotherapeutic agents (drug IC_50_, 24 h) or vehicle control, media were changed and cells were subsequently treated with DR ligands (0–40 ng/ml, 24 h). Viable cell number was determined by the Trypan blue exclusion assay. Results are presented as the mean of the viable cell number calculated as a percentage of untreated control cells±s.e.m. (*n*=9). Statistics indicate significant interactions between drugs and DR ligands, which is indicative of enhanced cell death. ^*^P⩽0.001, ^**^P⩽0.01, ^***^P⩽0.05.

Chemotherapeutics commonly used in the treatment of ESFT were also examined to determine whether they upregulated DR cell surface expression and enhanced DR ligand-induced cell death or whether this was a fenretinide-specific phenomenon. Etoposide increased expression of all DRs (Figure 4A, *P*⩽0.001), actinomycin D increased DR5 (*P*<0.001) and p75^NTR^ (*P*=0.03), whereas vincristine only upregulated FAS (*P*⩽0.001). On using the calculated IC_50_, doxorubicin did not significantly increase DR expression (Figure 4A). However, at 10 times the doxorubicin IC_50_ value, upregulation of all DRs was observed (data not shown, *P*=0.002). An increase in p75^NTR^ cell surface expression was detected when cells were treated with 10 times the vincristine IC_50_ (data not shown, *P*=0.001).

It is reported in the literature that etoposide induces DR upregulation through NF-*κ*B transcriptional upregulation (Gibson *et al*, 2000; Shetty *et al*, 2002; Woo *et al*, 2004; Mendoza *et al*, 2008). BAY 11-70892, an inhibitor of I*κ*B*α* phosphorylation and subsequent NF-*κ*B translocation to the nucleus, was used to elucidate whether this was the mechanism of upregulation in ESFT cells. BAY 11-7082 had no effect on DR upregulation (data not shown), suggesting that NF-*κ*B does not regulate etoposide-induced DR expression in ESFT cells. However, SB202190 pretreatment decreased both actinomycin D and etoposide-induced DR5 expression and actinomycin D-induced p75^NTR^ expression (data not shown). Furthermore, ASK1 kinase activity increased after treatment with etoposide and vincristine but not after treatment with doxorubicin or actinomycin D (Figure 4B). Actinomycin D failed to activate ASK1, although SB202190 prevented DR5 and p75^NTR^ upregulation, suggesting an alternative mechanism of upregulation. Collectively, these data are consistent with the hypothesis that both ASK1 and p38^MAPK^ are required for the chemotherapeutic-induced upregulation of certain DRs in some ESFT cells.

TC-32 cells were pretreated with chemotherapeutics which significantly increased DR expression at the cell surface, and subsequently treated with DR ligands. Both TRAIL and FasL were dependent on the presence of etoposide to induce cell death, as neither ligand induced death when used alone (Figure 4C, *P*⩽0.05). No statistical interaction was observed between etoposide and NGF combined treatment, despite a 15% decrease in viable cell number. Actinomycin D cotreatment with TRAIL (significant interaction of *P*⩽0.001), but not with NGF, resulted in additive cell death, whereas vincristine and FasL cotreatment did not further decrease viable cell number compared with treatment with vincristine alone (Figure 4C). The observations with cotreatments of FasL and vincristine, and NGF with etoposide or actinomycin D, suggest that high levels of DR expression and treatment with the cognate ligand might not always correlate with increased cytotoxicity. The effect of ligands and doxorubicin was therefore examined because no significant increase in DR expression was observed with this drug. No enhanced death was observed with doxorubicin and with either TRAIL or NGF compared with doxorubicin alone (Figure 4C).

## Discussion

**Figure 5:**
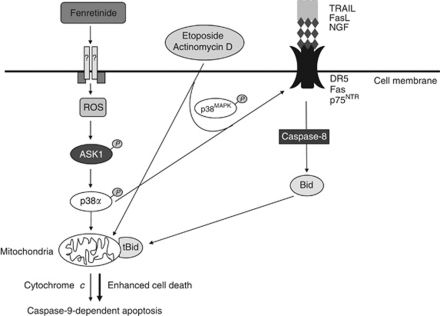
Combined treatment of fenretinide, etoposide or actinomycin D with TRAIL results in enhanced cell death. Treatment of ESFT cells with fenretinide leads to mitochondrial-dependent apoptosis through ROS generation, and ASK1-induced phosphorylation of p38^MAPK^. Fenretinide treatment also results in ASK1- and p38*α-*mediated upregulation of DR at the cell surface. Etoposide and actinomycin D had similar effects. Treatment with ligands to the cognate DR resulted in enhanced cell death, which was mediated through both caspase-8 and Bid cleavage (tBid=truncated Bid).

In this study, we demonstrate for the first time that cotreatment with fenretinide and DR ligands enhanced ESFT cell death compared with fenretinide alone, through the induction of both the mitochondrial and DR apoptotic pathways (Figure 5); DR ligands alone did not induce death. Fenretinide upregulated cell surface expression of DRs TRAIL, FAS and p75^NTR^ in an ASK1- and p38*α*-dependent manner. This is likely to be initiated through increased levels of ROS, as fenretinide-induced death (Myatt *et al*, 2005) and ASK1 phosphorylation are inhibited by antioxidants. ROS have previously been implicated in DR upregulation at the cell surface and in subsequent apoptosis in response to hydrogen peroxide (Kwon *et al*, 2008), 15-deoxy-delta12,14-prostaglandin J2 (Su *et al*, 2008), zerumbone (Yodkeeree *et al*, 2009) and arsenic trioxide (Woo *et al*, 2004), consistent with the hypothesis that induction of DR expression is effected through the generation of ROS. Induction of DR4 protein expression by bortezomib (Nakamura *et al*, 2007) and downregulation of DR5 cell surface expression by HDACi (Sonnemann *et al*, 2007) have previously been reported in ESFT cells.

p38^MAPK^ induces p75^NTR^ protein expression in ESFT cells in response to bFGF (Williamson *et al*, 2004) and p75^NTR^ mRNA and protein expression in prostate cancer cells by inflammatory agents (Quann *et al*, 2007; Khwaja *et al*, 2008). Furthermore, zerumbone induces DR4 but not DR5 protein expression through a p38^MAPK^-dependent mechanism in colon cancer cells (Yodkeeree *et al*, 2009). However, the p38^MAPK^ isoform involved in these processes has not been identified. This is likely to be important for therapeutic interventions exploiting p38^MAPK^, as four isoforms exist that regulate varied biological processes (Coulthard *et al*, 2009). Induction of death through specific p38^MAPK^ isoforms that do not result in unacceptable side effects and toxicities will be more attractive than if isoforms that are implicated in toxicity are involved. The mechanism by which ASK1 and p38^MAPK^ upregulate DR in ESFT cells remains to be elucidated. Upregulation of both mRNA and cell surface expression of TRAIL DR by agents such as etoposide, proteasome inhibitors and arsenic trioxide is frequently reported to be mediated by p53 (Chen *et al*, 2008) and/or NF-*κ*B (Woo *et al*, 2004; Chen *et al*, 2008; Mendoza *et al*, 2008; Song *et al*, 2008). However, we have found that DR upregulation in ESFT cells is not dependent on EWS–ETS fusion type, p53, p16 (Brownhill *et al*, 2007) or NF-*κ*B status, suggesting that the manner of DR upregulation is cell type and stimulus dependent. DR5 is reported to be upregulated by the transcription factor CHOP in several cell lines by different stimuli, for example, 15-deoxy-delta12, 14-prostaglandin J2 (Su *et al*, 2008), proteosome inhibitors (Hetschko *et al*, 2008) and endoplasmic reticulum stress (Yamaguchi and Wang, 2004). One report has shown that fenretinide and TRAIL enhance apoptosis through CHOP-dependent DR5 upregulation (Kouhara *et al*, 2007). No differences in DR4 and FAS mRNA levels were reported; hence studies on these receptors were discontinued. These observations are consistent with our cDNA array data but importantly we discovered that fenretinide upregulated these receptors at the cell surface. Further investigation into how p38*α* upregulates DR at the surface of ESFT cells is required. This may be mediated by CHOP because it has previously been implicated in fenretinide-induced death (Hail *et al*, 2006) and is a known p38^MAPK^ substrate (Coulthard *et al*, 2009).

p38^MAPK^ inhibitors failed to reduce fenretinide-induced DR expression to basal levels, suggesting that other factors may also upregulate DR. c-Jun N-terminal kinase is an additional ASK1 substrate that is activated by fenretinide in certain cell types (Kang *et al*, 2008; Appierto *et al*, 2009). Several groups report dual activation of p38^MAPK^ and c-Jun N-terminal kinase (Osone *et al*, 2004; Kim *et al*, 2006; Woo *et al*, 2009), suggesting that both MAPK may be required for fenretinide-induced DR upregulation in ESFT cells.

Cotreatment with fenretinide and DR ligands resulted in synergistic cell death through caspase-8 and Bid-dependent pathways, although confirmation that these are effectors of cell death requires validation through siRNA or inhibitor studies. This amplified death above that induced by either agent alone reflected activation through both the extrinsic (DR; Ashkenazi, 2008) and intrinsic (fenretinide; Myatt *et al*, 2005) apoptotic pathways (Figure 5). Fenretinide is also reported to activate caspase-8 and caspase-9 in glioblastoma (Das *et al*, 2008), meningioma and ovarian carcinoma (Cuello *et al*, 2004; Hail *et al*, 2006). We also demonstrated that etoposide and vincristine induce DR upregulation at the cell surface through ASK1 and p38^MAPK^, which subsequently results in enhanced death when cells are treated with DR ligands (although these effects were more modest than those observed with fenretinide). These observations support previously published data that etoposide increases DR5 mRNA and protein expression (Gibson *et al*, 2000), although they contradict studies in which vincristine had no effect on FAS mRNA expression in HL-60 leukaemic cells (Thomadaki *et al*, 2009) and the synergistic effect of actinomycin D through downregulation of XIAP with no effect on TRAIL DR expression (Ng *et al*, 2002). Doxorubicin has also been shown to induce DR5 cell surface expression in other cell types (Yoshida *et al*, 2003), suggesting that the effects of chemotherapeutics on DR expression is cell-line dependent. However, only actinomycin D, TRAIL and etoposide, with either TRAIL or FasL, enhanced cell death compared with either agent alone, consistent with the observation that high levels of DR expression do not necessarily correlate with ligand-induced cytotoxicity (Georgakis *et al*, 2005). In all drug combinations, a 20–40% cell population remained refractory to treatment. This may be explained by the presence of cancer stem cell like cells which are hypothesised to be responsible for resistance to therapy and tumour relapse (Hemmings, 2010).

Importantly, fenretinide did not increase DR expression in non-malignant MSC and NHUC. Furthermore, fenretinide, TRAIL or the combination of both agents was not cytotoxic to either of these non-malignant cell lines, indicating that the combination of fenretinide and TRAIL may have minimal toxicity. Interestingly, FAS was downregulated by fenretinide, and both DcRs were strongly increased in NHUC. We also observed that fenretinide upregulated TRAIL DcR in ESFT cells, which supports previous data in which TRAIL DcRs are upregulated alongside DR by apoptotic stimuli such as UV (Maeda *et al*, 2001), doxorubicin (Yoshida *et al*, 2003) and oxaplatin (Toscano *et al*, 2008). Collectively, these observations suggest a possible mechanism to evade apoptosis, the higher DcR to DR ratio being likely to drive cells towards a survival pathway. Cytoprotective roles for DcR have previously been elucidated and are attributed to DcR-mediated stimulation of the Akt, ERK (Secchiero *et al*, 2003) and NF-*κ*B (Degli-Esposti *et al*, 1997) survival pathways, competitive binding of TRAIL to DcR1 inhibiting DR-induced DISC formation and DcR2 forming ligand-independent complexes with DR5 at the DISC to inhibit DR4 corecruitment and caspase activation (Ashkenazi, 2008).

Our work demonstrates that the combination of fenretinide and DR ligands, actinomycin D and TRAIL and etoposide used in combination with either TRAIL or FasL may be advantageous for the treatment of ESFT cells. As DR ligands transduce apoptotic signals through different pathways to those of chemotherapy and irradiation, these combinations may augment patient response to either drug alone and so be more effective. The combination of fenretinide (Garaventa *et al*, 2003; Villablanca *et al*, 2006; Formelli *et al*, 2008) and TRAIL (Ashkenazi, 2008) is an attractive therapeutic strategy because both agents are well tolerated in clinical trials and non-malignant cells are resistant to their cytotoxic effects, unlike many commonly used agents that are used in combination with TRAIL. Furthermore, both agents induce cytotoxicity independently of p53 (Hail *et al*, 2006; Ashkenazi, 2008), which is important when p53 mutations are common in certain cancer types and many conventional treatments such as irradiation and DNA-damaging drugs rely on p53 to induce cell death.

The combination of FasL with fenretinide or etoposide may also have therapeutic potential. However, at present, this is not a viable combination in the clinic because FAS monoclonal antibodies have shown hepatotoxicity in preclinical trials (Ashkenazi, 2008). However, APO010 (a second generation FasL from TopoTarget) is a recombinant mega-FasL that displays anticancer activities both *in vitro* and in xenograft models of human cancer. A dose-escalation phase I trial is currently recruiting participants with solid tumours to examine APO010 toxicity (ClinicalTrials.gov Identifier: NCT00437736).

Two strategies are currently being explored for the clinical use of TRAIL: recombinant human preparations (Genentech, San Francisco, CA, USA) or monoclonal antibodies to DR4 (Mapatumumab, Human Genome Sciences, Rockville, MD, USA) and DR5 (Amgen, Daiichi Sankyo (Munich, Germany), Human Genome Sciences, Genentech and Novartis, Basel, Switzerland). Both show promise in clinical trials when used as monotherapies and in combination with agents such as chemotherapeutics, XIAP inhibitors, proteasome inhibitors, HDACi, natural products and BH3 mimetics (Ashkenazi, 2008). As mentioned previously, Mapatumumab only induced limited *in vitro* cytotoxicity and growth inhibition in ESFT xenograft tumour panels (Smith *et al*, 2009). Low DR4 mRNA expression was observed, in concordance with our results, suggesting that combination therapies of TRAIL receptor agonists with fenretinide or chemotherapeutic drugs would be a better therapeutic regime for the treatment of paediatric tumours because of the upregulation of DR or downregulation of antiapoptotic factors.

In summary, the synergistic cell death observed with the combined treatment of fenretinide with DR ligands is mediated through ASK1- and p38*α*-induced upregulation of DR at the cell surface. In contrast, only certain chemotherapeutic drugs upregulated DR at the cell surface, which did not always correspond to enhanced cell death when cells were cotreated with the cognate DR ligand. These data suggest that fenretinide in combination with TRAIL may be a particularly attractive therapeutic strategy, as both agents are well tolerated in clinical trials and both induce death through separate apoptotic pathways that may overcome chemoresistance.
