# Supplementary material for: Fenretinide-dependent upregulation of death receptors through ASK1 and p38α enhances death receptor ligand-induced cell death in Ewing's sarcoma family of tumours
Source: Br J Cancer. 2010 Sep 28;103(9):1380–90. doi: 10.1038/sj.bjc.6605896 (PMC2990598; doi:10.1038/sj.bjc.6605896)
Supplement: Supplementary Figures 1–4 [file 6605896x1.ppt]

## Slide 1
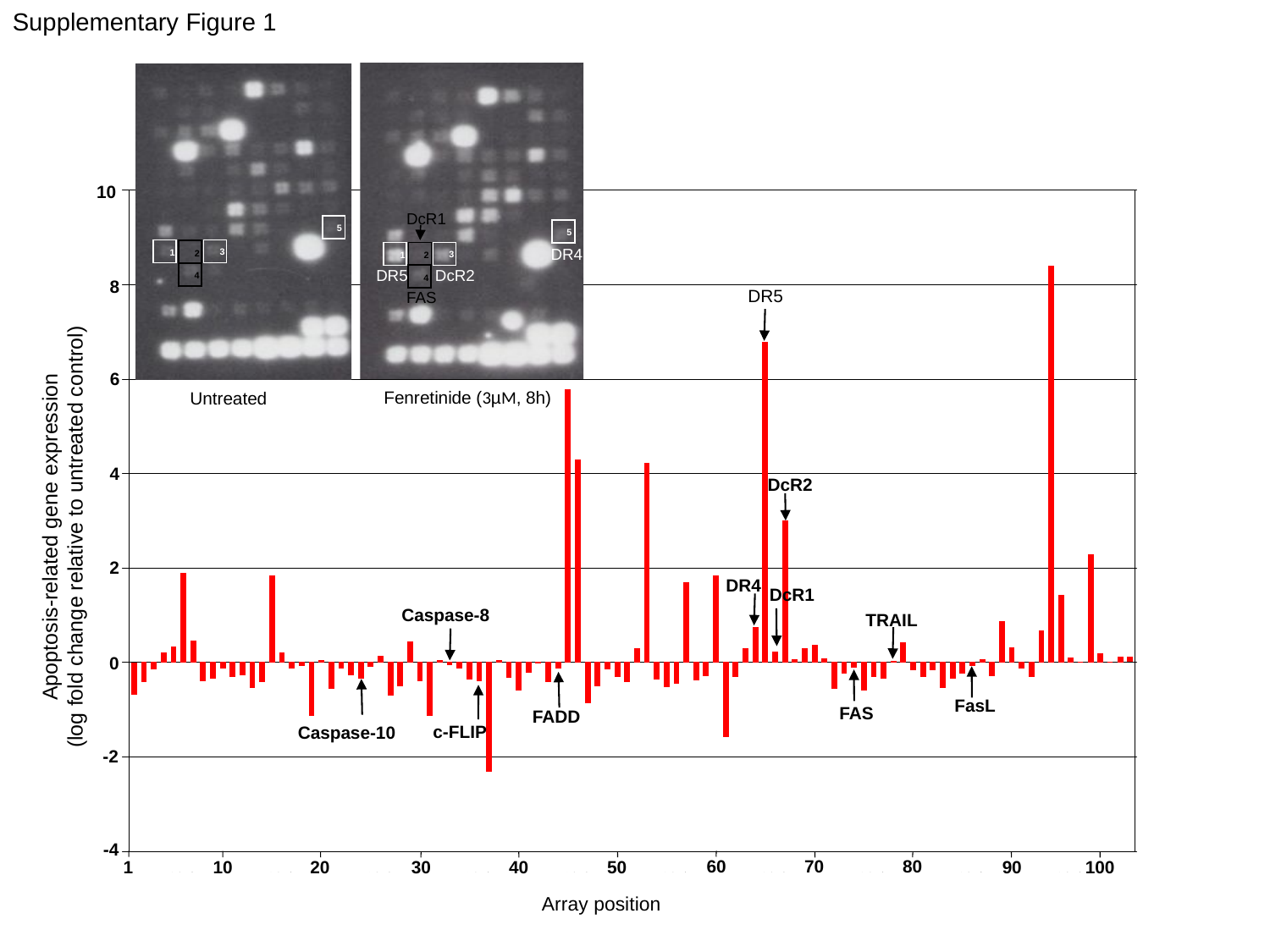

Supplementary Figure 1
DcR1
5
5
3
DR4
1
2
3
2
1
DR5
DcR2
4
4
FAS
Fenretinide (3µM, 8h)
Untreated
DR5
10
8
6
4
DcR2
 Apoptosis-related gene expression
(log fold change relative to untreated control)
2
DR4
DcR1
Caspase-8
TRAIL
0
FasL
FAS
FADD
c-FLIP
Caspase-10
-2
-4
60
70
80
1
10
20
30
40
50
90
100
Array position

## Slide 2
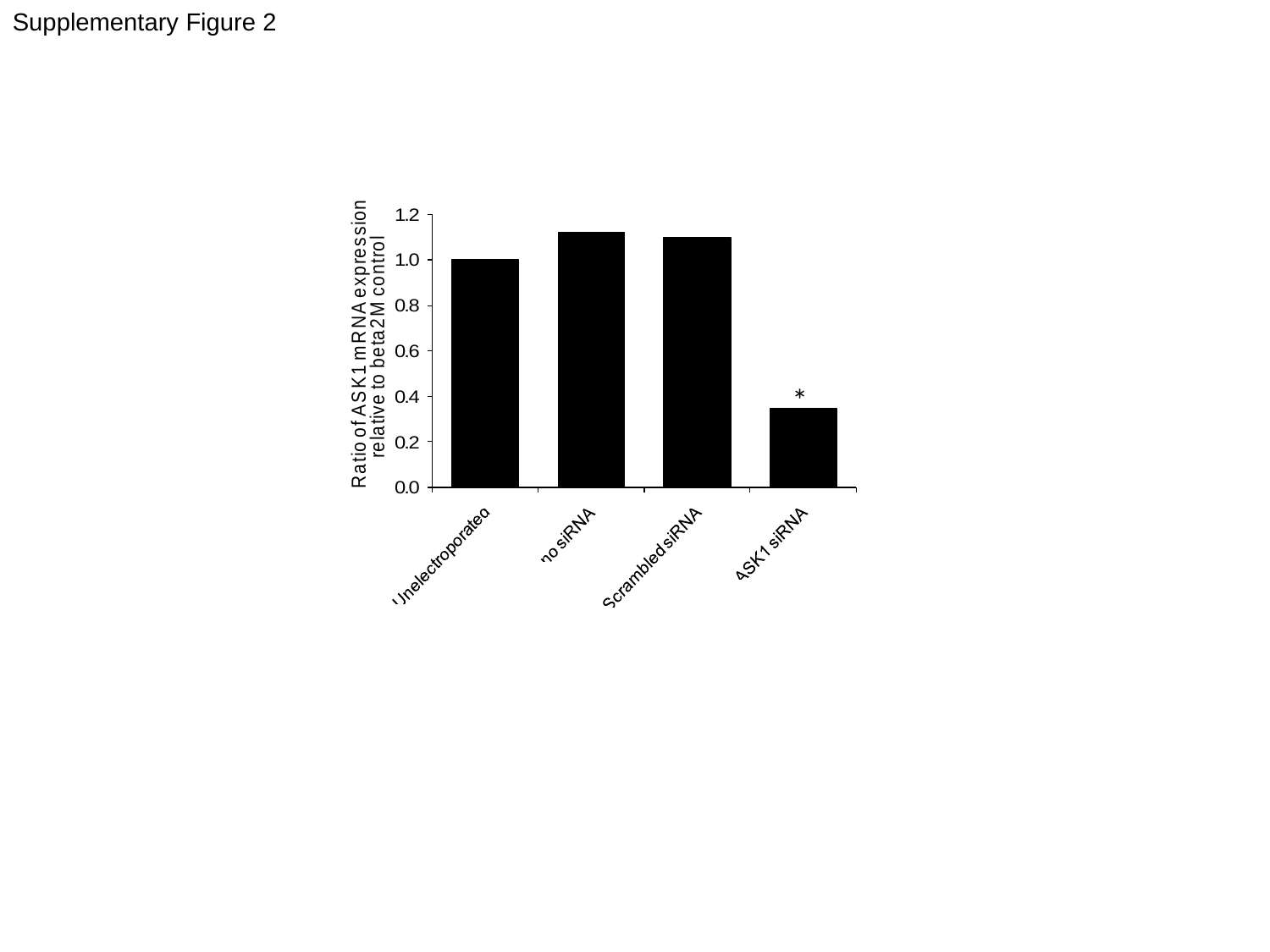

Supplementary Figure 2
*

## Slide 3
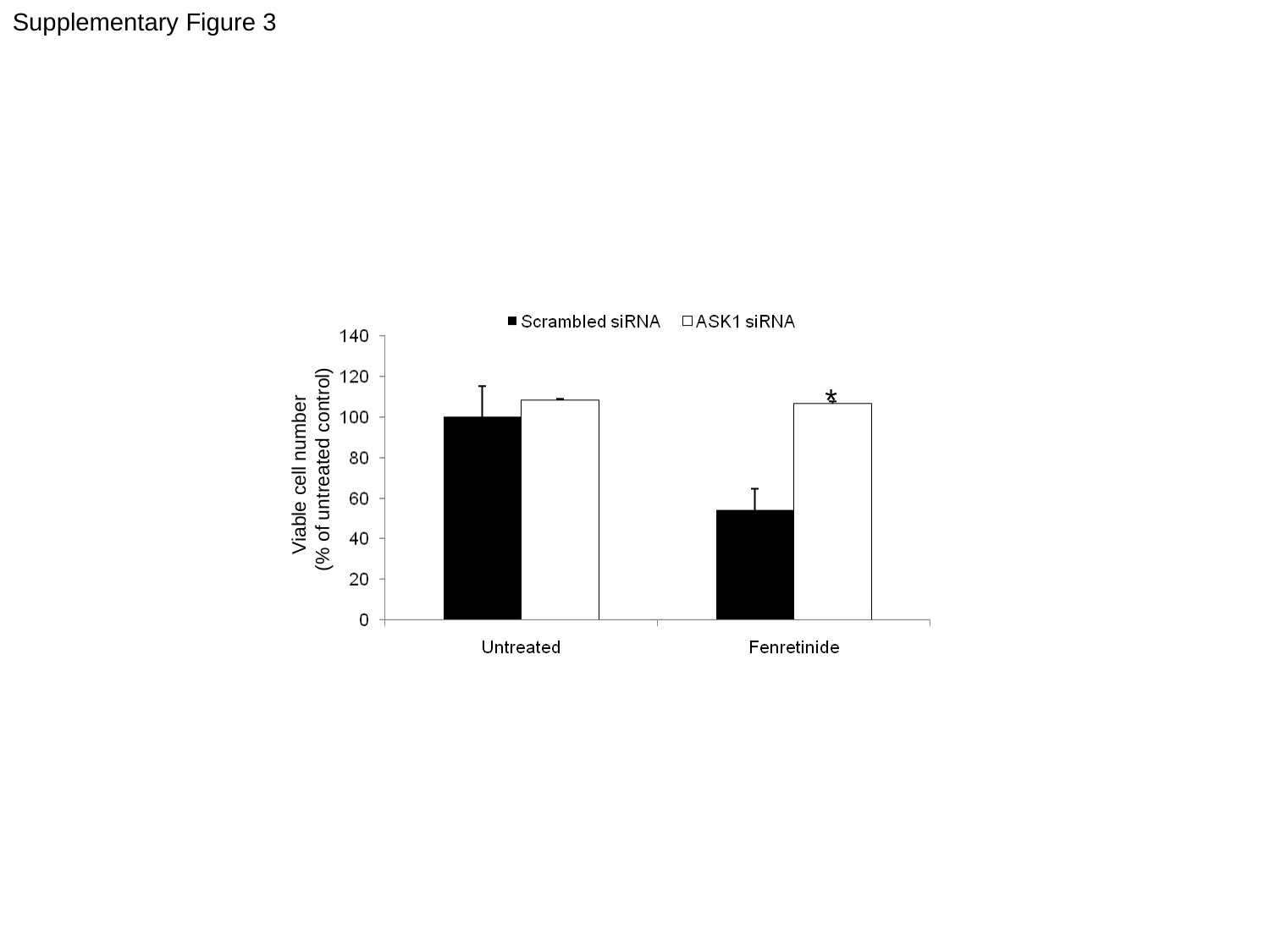

Supplementary Figure 3
*
Viable cell number
 (% of untreated control)

## Slide 4
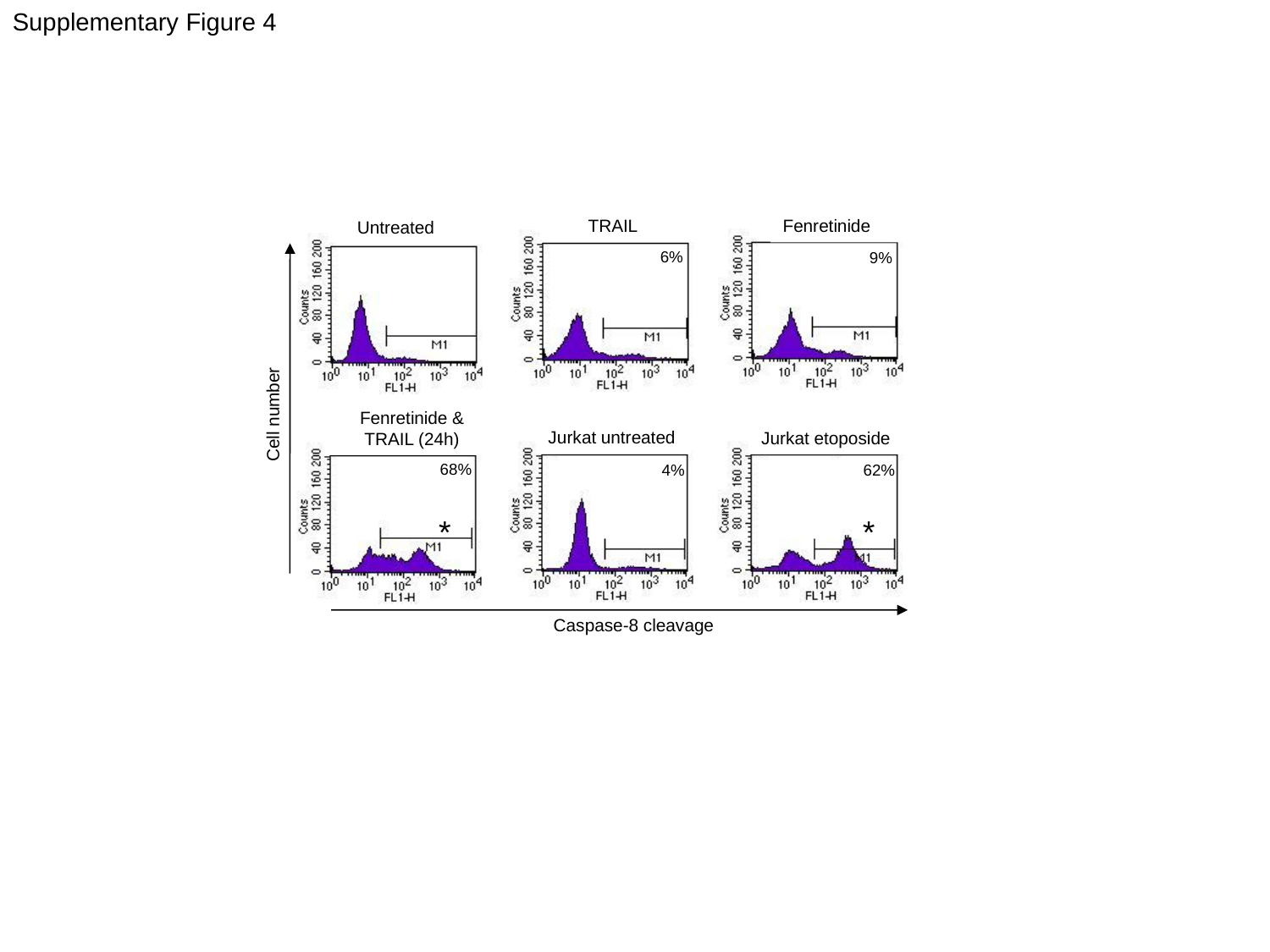

Supplementary Figure 4
 TRAIL
Fenretinide
 Untreated
6%
Cell number
Fenretinide & TRAIL (24h)
Jurkat untreated
Jurkat etoposide
68%
4%
62%
*
Caspase-8 cleavage
*
*
9%
