# Supplementary material for: Fenretinide-dependent upregulation of death receptors through ASK1 and p38α enhances death receptor ligand-induced cell death in Ewing's sarcoma family of tumours
Source: Br J Cancer. 2010 Sep 28;103(9):1380–90. doi: 10.1038/sj.bjc.6605896 (PMC2990598; doi:10.1038/sj.bjc.6605896)
Supplement: Supplementary Figure Legends [file 6605896x2.doc]

**Supplementary Figure Legends**

**Supplementary Figure 1**

TC-32 cells were treated with fenretinide (3μM, 8h) or vehicle control and total RNA was extracted and used to generate a biotin-labelled cDNA library, which was hybridised to an apoptosis-specific cDNA array. Graph shows expression levels of all apoptosis-related genes relative to untreated control (log fold change; see Supplementary Table 2 for complete list of genes and array position). Insert) Array image from untreated and fenretinide treated TC-32 cells. White boxes indicate an increase in cDNA expression with fenretinide treatment, whereas black boxes indicate no expression change.

**Supplementary Figure 2**

TC-32 cells were electroporated with siRNA (500nM) directed against ASK1 (a pool of three individual ASK1 siRNAs) or scrambled siRNA. Cells were harvested 48h post electroporation and ASK1 mRNA expression levels were determined by quantitative RT-PCR. Results are presented as the mean ASK1 mRNA expression relative to the housekeeping gene β-2M and normalised to the unelectroporated sample ± SEM (n=6). *p≤0.001.

**Supplementary Figure 3**

TC-32 cells were electroporated with scrambled or ASK1 siRNA (500nM) and treated with fenretinide (3μM, 24h) 24h post-electroporation. Viable cell number was determined by the trypan blue exclusion assay. Results are presented as the mean of the viable cell number calculated as a percentage of untreated control cells ± SEM (n=6). * p<0.01.

**Supplementary Figure 4**

TC-32 cells were pre-treated with fenretinide (1.5µM, 16h) prior to TRAIL treatment (40ng/ml, 24h) and caspase-8 activity was detected by flow cytometry. Representative flow cytometry images (n=9) depicting caspase-8 cleavage are shown. Percentages given are the number of cells in which caspase-8 is cleaved and subsequently detected in the M1 region. Untreated and etoposide (30μM, 24h) treated Jurkat cells served as negative and positive control samples respectively.
