# Supplementary material for: Fenretinide-dependent upregulation of death receptors through ASK1 and p38α enhances death receptor ligand-induced cell death in Ewing's sarcoma family of tumours
Source: Br J Cancer. 2010 Sep 28;103(9):1380–90. doi: 10.1038/sj.bjc.6605896 (PMC2990598; doi:10.1038/sj.bjc.6605896)
Supplement: Supplementary Table 1 [file 6605896x3.doc]

**Supplementary Table 1:** Cell lines and culture conditions used

Abbreviations: ATCC®: **American Type Culture Collection, ESFT: Ewing’s sarcoma family of tumours, MSC: Mesenchymal stem cells, NHU: Normal human urothelial. # Leeds Institute of Molecular Medicine, Leeds, UK. * Cultured on Primeria plastic, all other cell lines were cultured on Falcon** (Fahrenheit, Leeds, UK) plastic.

References

1. Myatt SS, Redfern CP, Burchill SA. p38MAPK-Dependent sensitivity of Ewing's sarcoma family of tumors to fenretinide-induced cell death. Clin Cancer Res 2005; 11:3136-48.
2. Chapman EJ, Hurst CD, Pitt E, Chambers P, Aveyard JS, Knowles MA. Expression of hTERT immortalises normal human urothelial cells without inactivation of the p16/Rb pathway. Oncogene 2006; 25:5037-45.

**Note:** ESFT cell lines reflect the different sites, EWS fusion type (confirmed by fluorescence in situ hybridization and RT-PCR), p53 and p16 heterogeneity of primary ESFT (9, 17). All ESFT lines express CD99 at the cell surface (confirmed by immunohistochemistry and flow cytometry). All cell lines are yeast, bacterial and mycoplasma-free (determined every four months using the EZ-PCR mycoplasma test kit (Geneflow, Fradley, UK) according to the manufacturer’s instructions).
