# Supplementary material for: Fenretinide-dependent upregulation of death receptors through ASK1 and p38α enhances death receptor ligand-induced cell death in Ewing's sarcoma family of tumours
Source: Br J Cancer. 2010 Sep 28;103(9):1380–90. doi: 10.1038/sj.bjc.6605896 (PMC2990598; doi:10.1038/sj.bjc.6605896)
Supplement: Supplementary Table 2 [file 6605896x4.doc]

| Position | **UniGene** | **Genebank** | **Gene name** |
| --- | --- | --- | --- |
| 1 | Hs.77579 | NM_001160 | Apoptotic protease activating factor |
| 2 | Hs.71869 | NM_013258 | Apoptosis-associated speck-like protein |
| 3 | Hs.194382 | NM_000051 | Ataxia telangiectasia mutated |
| 4 | Hs.76366 | U66879 | BCL2-antagonist of cell death |
| 5 | Hs.93213 | AA923639 | BCL2-antagonist/killer 1 |
| 6 | Hs.159428 | L22474 | BCL2-associated X protein |
| 7 | Hs.193516 | NM_003921 | B-cell CLL/lymphoma 10 |
| 8 | Hs.79241 | M14745 | B-cell CLL/lymphoma 2 |
| 9 | Hs.227817 | NM_004049 | BCL2-related protein A1 |
| 10 | Hs.305890 | Z23115 | BCL2-like 1 |
| 11 | Hs.202657 | AF032458 | BCL2-like 11 (apoptosis facilitator) |
| 12 | Hs.75244 | U59747 | BCL2-like 2 |
| 13 | Hs.155419 | U34584 | BCL2-interacting killer |
| 14 | Hs.79019 | NM_004536 | Baculoviral IAP repeat-containing 1 |
| 15 | Hs.289107 | U45879/U37547 | Inhibitor of apoptosis protein 2 |
| 16 | Hs.127799 | U37546 | Inhibitor of apoptosis protein 1 |
| 17 | Hs.172777 | NM_001167 | X-linked IAP |
| 18 | Hs.1578 | U75285 | Survivin |
| 19 | Hs.250646 | AF265555 | IAP repeat-containing 6 |
| 20 | Hs.2243 | Z33998 | B lymphoid tyrosine kinase |
| 21 | Hs.79428 | AF002697 | BCL2/adenovirus E1B 19kD-interacting protein 3 |
| 22 | Hs.307355 | AF089746 | BCL2-related ovarian killer |
| 23 | Hs.2490 | M87507 | Caspase 1 |
| 24 | Hs.5353 | U60519 | Caspase 10 |
| 25 | Hs.137587 | NM_003723 | Caspase 13 |
| 26 | Hs.248226 | NM_012114 | Caspase 14 |
| 27 | Hs.108131 | U13021 | Caspase 2 |
| 28 | Hs.74552 | NM_004346 | Caspase 3 |
| 29 | Hs.74122 | U28014 | Caspase 4 |
| 30 | Hs.3257 | U28015 | Caspase 5 |
| 31 | Hs.3280 | U20537 | Caspase 6 |
| 32 | Hs.9216 | U67320 | Caspase 7 |
| 33 | Hs.19949 | NM_001228 | Caspase 8 |
| 34 | Hs.122843 | AF154415 | CASP8 associated protein 2/CASPER/  c-FLIP |
| 35 | Hs.100641 | U60521 | Caspase 9 |
| 36 | Hs.195175 | AF010127 | CASP8 and FADD-like apoptosis regulator |
| 37 | Hs.20295 | AF016582 | CHK1 (checkpoint, S.pombe) homolog |
| 38 | Hs.249129 | NM_001279 | Cell death-inducing DFFA-like effector a |
| 39 | Hs.288835 | NM_014430 | Cell death-inducing DFFA-like effector b |
| 40 | Hs.155566 | NM_003805 | CASP2 and RIPK1 domain containing adaptor with death domain |
| 41 | Hs.129208 | NM_014326 | Death-associated protein kinase 2 |
| 42 | Hs.105658 | NM_004401 | DNA fragment factor-45 |
| 43 | Hs.133089 | NM_004402 | DNA fragmentation factor, 40 kD, beta subunit |
| 44 | Hs.86131 | NM_003824 | FADD |
| 45 | Hs.80409 | M60974 | DNA-damage-inducible transcript 1 |
| 46 | Hs.87247 | NM_003806 | Harakiri |
| 47 | Hs.152983 | NM_004507 | HUS1 (S. pombe) checkpoint homolog |
| 48 | Hs.168159 | AF173003 | Apoptosis regulator |
| 49 | Hs.36 | D12614 | Lymphotoxin-alpha |
| 50 | Hs.890 | NM_002341 | Lymphotoxine-beta |
| 51 | Hs.1116 | L04270 | Homo sapiens lymphotoxin beta receptor |
| 52 | Hs.86386 | L08246 | Myeloid cell leukemia sequence 1 |
| 53 | Hs.170027 | Z12020 | Mouse double minute |
| 54 | Hs.82116 | NM_002468 | Myeloid differentiation primary response gene (88) |
| 55 | Hs.19405 | NM_006092 | Caspase recruitment domain 4 |
| 56 | Hs.278439 | AF043244 | Nucleolar protein 3 |
| 57 | Hs.74368 | X69910 | p63 mRNA for transmembrane protein |
| 58 | Hs.146329 | NM_007194 | Protein kinase Chk2 |
| 59 | Hs.296327 | U50062 | Receptor-interacting serine-threonine kinase 1 |
| 60 | Hs.103755 | AF078530 | Receptor-interacting serine-threonine kinase 2 |
| 61 | Hs.1608 | L07493 | Replication protein A3 |
| 62 | Hs.146847 | U59863 | TRAF family member-associated NFKB activator |
| 63 | Hs.241570 | X01394 | Tumor necrosis factor alpha |
| 64 | Hs.249190 | U90875 | TRAIL receptor 1/DR4 |
| 65 | Hs.51233 | AF016266 | TRAIL receptor 2/DR5 |
| 66 | Hs.119684 | AF016267 | TRAIL receptor 3/DcR1 |
| 67 | Hs.129844 | AF021232 | TRAIL receptor 4/DcR2 |
| 68 | Hs.180338 | U74611 | TRAMP/Apo-3/DR3 |
| 69 | Hs.279899 | U81232 | Human tumor necrosis factor receptor-like gene 2 |
| 70 | Hs.159 | M33294 | Tumor necrosis factor receptor 1 |
| 71 | Hs.256278 | NM_001066 | Tumor necrosis factor receptor 2 |
| 72 | Hs.129780 | X75962 | OX40 |
| 73 | Hs.25648 | NM_001250 | CD40 |
| 74 | Hs.82359 | X63717 | FAS |
| 75 | Hs.180841 | M63928 | CD27 receptor |
| 76 | Hs.1314 | NM_001243 | CD30 ligand receptor |
| 77 | Hs.73895 | U03397 | CD137 |
| 78 | Hs.83429 | U37518 | TRAIL |
| 79 | Hs.115770 | AF19047 | Osteoprotegerin ligand/TRANCE |
| 80 | Hs.26401 | AF055872 | Apo3/DR3 ligand |
| 81 | Hs.54673 | NM_003808 | April |
| 82 | Hs.129708 | AF036581 | LIGHT/HVEM-L |
| 83 | Hs.181097 | D90224 | OX40 ligand |
| 84 | Hs.652 | L07414 | CD40 ligand |
| 85 | Hs.2007 | U08137 | Fas ligand |
| 86 | Hs.99899 | L08096 | CD27 ligand |
| 87 | Hs.1313 | L09753 | CD30 ligand |
| 88 | Hs.1524 | U03398 | TNFSF9/4-1BB |
| 89 | Hs.1846 | M14694 | p53 |
| 90 | Hs.2134 | NM_005658 | TNF receptor-associated factor (TRAF) 1 |
| 91 | Hs.200526 | U12597 | TRAF 2 |
| 92 | Hs.297660 | NM_003300 | TRAF 3 |
| 93 | Hs.8375 | AF082185 | TRAF 4 |
| 94 | Hs.29736 | AB000509 | TRAF 5 |
| 95 | Hs.90957 | NM_004620 | TRAF 6 |
| 96 | Hs.21254 | U77845 | TRAF interacting protein |
| 97 | N/A | L08752 | PUC18 Plasmid DNA |
| 98 | N/A | L08752 | PUC18 Plasmid DNA |
| 99 | N/A | L08752 | PUC18 Plasmid DNA |
| 100 | Blank | Blank | Blank |
| 101 | Blank | Blank | Blank |
| 102 | Blank | Blank | Blank |
| 103 | Hs.169476 | M33197 | Glyceraldehyde-3-phosphate dehydrogenase |
| 104 | Hs.169476 | M33197 | Glyceraldehyde-3-phosphate dehydrogenase |
| 105 | Hs.342389 | NM_021130 | Cyclophilin A |
| 106 | Hs.342389 | NM_021130 | Cyclophilin A |
| 107 | Hs.342389 | NM_021130 | Cyclophilin A |
| 108 | Hs.342389 | NM_021130 | Cyclophilin A |
| 109 | Hs.119122 | NM_012423 | Ribosomal protein L13a |
| 110 | Hs.119122 | NM_012423 | Ribosomal protein L13a |
| 111 | Hs.288061 | X00351 | Beta Actin |
| 112 | Hs.288061 | X00351 | Beta Actin |

### Supplementary Table 2: Gene names and array position for the GEArray® Q series human apoptosis gene array
